# Enhancing breast cancer classification via histopathological image analysis: Leveraging self-supervised contrastive learning and transfer learning

**DOI:** 10.1016/j.heliyon.2024.e24094

**Published:** 2024-01-09

**Authors:** Faisal Bin Ashraf, S.M. Maksudul Alam, Shahriar M. Sakib

**Affiliations:** a Department of Computer Science and Engineering, University of California, Riverside, 92521, CA, USA; b Marlan and Rosemary Bourns College of Engineering, University of California, Riverside, 92521, CA, USA

**Keywords:** Breast cancer classification, Histopathology image, Transfer learning, Contrastive learning, Medical image processing

## Abstract

Breast cancer, a significant threat to women's health, demands early detection. Automating histopathological image analysis offers a promising solution to enhance efficiency and accuracy in diagnosis. This study addresses the challenge of breast cancer histopathological image classification by leveraging the ResNet architecture, known for its depth and skip connections. In this work, two distinct approaches were pursued, each driven by unique motivations. The first approach aimed to improve the learning process through self-supervised contrastive learning. It utilizes a small subset of the training data for initial model training and progressively expands the training set by incorporating confidently labeled data from the unlabeled pool, ultimately achieving a reliable model with limited training data. The second approach focused on optimizing the architecture by combining ResNet50 and Inception module to get a lightweight and efficient classifier. The dataset utilized in this work comprises histopathological images categorized into benign and malignant classes at varying magnification levels (40X, 100X, 200X, 400X), all originating from the same source image. The results demonstrate state-of-the-art performance, achieving 98% accuracy for images magnified at 40X and 200X, and 94% for 100X and 400X. Notably, the proposed architecture boasts a substantially reduced parameter count of approximately 3.6 million, contrasting with existing leading architectures, which possess parameter sizes at least twice as large.

## 1 Introduction

**Figure 1:**
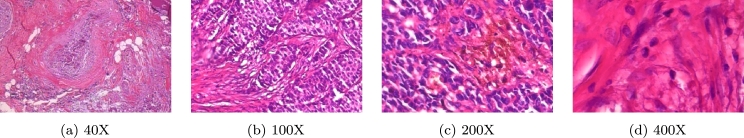
Histopathology images with cancer nuclei at different magnification levels.

Breast cancer ranks as the primary cause of cancer-related deaths for women worldwide, trailing only lung cancer [1]. Most of the existing methods of diagnosing breast cancer involve a manual examination of biopsy samples under a microscope [2]. The methods sometimes can be laborious and susceptible to human errors [3][4]. Generally, histopathology images, as shown in Fig. 1(a-d), are used to identify different types of cell nuclei and their architectural patterns [5]. Histopathologists visually analyze the images to find out the regularities in cell shapes and tissue distributions as cancerous regions and assess the degree of malignancy [6][7]. Although diagnostic imaging technologies have advanced, pathologists still rely on visual inspection of histological samples for accurate diagnosis, grading, and staging [4]. Manual diagnosis of cancer from histopathological images can be challenging due to the potential inaccuracies of detection caused by the limitations of humans.

To enhance the efficiency and accuracy of analyzing histopathological images, computer-vision-aided diagnosis systems can be introduced effectively. Deep learning-based automated approaches already exist for distinguishing between malignant and benign cancer images [8]. These approaches excel, particularly in early-stage cancer diagnosis, thanks to the capabilities of deep learning. At the core of deep learning architecture are convolutional neural networks (CNNs), a specialized feed-forward neural network design that incorporates convolution and pooling layers as fully connected hidden layers [9]. CNNs have since become cutting-edge solutions for tasks such as large-scale object classification and object detection [10][11]. In the context of breast cancer histopathology image classification, CNNs have demonstrated successful applications in previous studies. Single-task and multi-task CNN models were introduced for the classification of the BreakHis Histopathological dataset [12]. While the positive aspect of the work is its independence from histopathological images, the performance in terms of classification accuracy is somewhat lacking.

To train CNNs effectively, an approach used the sliding window mechanism for patch extraction, facilitating efficient processing [13]. However, due to the use of relatively small patch sizes, specifically 32x32 and 64x64, the generated patches may suffer from a loss of useful information and context. In the study involving a sequential framework and deep feature extraction from a fine-tuned DenseNet model trained on the BreakHis dataset, the extracted deep features were subsequently employed as input data for an XGBoost classifier [14]. Nevertheless, relying solely on validation data for determining the cutoff thresholds of each layer used for classification confidence could introduce inaccuracies that may significantly impact the results. Transfer learning and deep feature extraction methods with CNN models (AlexNet and VGG16) were employed for early breast cancer detection in another study [15]. Their experiments demonstrated the superiority of transfer learning over deep feature extraction and SVM classification in achieving higher accuracy. In a recent study, VGGIN-Net, a novel deep neural network architecture is proposed for breast cancer classification [16]. This approach involved combining layers from a pre-trained VGG16 model with a straightforward Inception block module, ultimately achieving enhanced performance through block-wise fine-tuning. Nevertheless, their achievement of strong performance in classification comes at the cost of a complex model, which comprises approximately 8 million parameters.

Given the recent strides in image processing and machine learning for breast cancer diagnosis through histopathology images, the demand for more precise diagnostic systems is paramount. Utilizing the ResNet architecture is motivated by its depth and skip connections, which can capture intricate patterns and enhance diagnostic accuracy [17]. In this work, we have pursued two distinct approaches driven by separate motivations. The first approach seeks to enhance the learning process by incorporating self-supervised contrastive learning. Conversely, the second approach concentrates on optimizing the architecture through the fusion of ResNet50 and a simple inception model. The objectives of this work are:

• Classify malignant images from histopathological images.
• Investigate contrastive learning approach.
• Apply transfer learning for effective classification.
• Design a lightweight architecture with improved performance.

## 2 Materials and methods

### 2.1 Dataset

In this work, the publicly available BreakHis dataset, consisting of 7,909 high-resolution RGB breast histopathological images [2] has been used. The dataset categorizes images into different sub-collections based on their magnification factors (40X, 100X, 200X, and 400X). All the images in the dataset are of the dimension of 700x460 pixels and are categorized into two classes: Benign and Malignant tumor. Additionally, both the Benign and Malignant tumor images are categorized into subtypes depending on their appearance under the microscope. Table 1 presents the sample distribution for each magnification factor. Notably, the dataset exhibits a class imbalance, with 2,480 benign samples and 5,429 malignant samples.

**Table 1:** The number of histology image samples per class.

Class	Sub-Class	Magnification factor			
		40X	100X	200X	400X
Benign	Adenosis	114	113	111	106
	Fibroadenoma	193	260	264	137
	Phyllodes tumor	149	150	140	130
	Tubular Adenona	109	121	108	115

Malignant	Carcinoma	864	903	896	788
	Lobular carcinoma	156	170	163	137
	Mucinous carcinoma	205	222	196	169
	Papillary Carcinoma	145	142	135	138

#### 2.1.1 Data preprocessing

The dataset for breast cancer detection was structured into four categories: 400x, 200x, 100x, and 40x, each containing both benign and malignant class images. To ensure compatibility with the proposed approaches, normalization was performed based on individual mean and standard deviation. The images were resized to 350x240 and then cropped to achieve a final size of 224x224, ensuring alignment with the intended training dimensions. To introduce variations, four augmentation techniques were applied to the data: horizontal and vertical shifts, flips, and rotations.

### 2.2 Methodology

The proposed work mainly focuses on two concerns: finding an efficient learning method, and a novel and effective architecture for a deep learning approach. To find an efficient learning method, a self-supervised contrastive learning approach has been presented in 2.2.1. On the other hand, a novel and effective architecture has been proposed combining different blocks of ResNet and Inception modules in 2.2.3.

#### 2.2.1 Self-supervised contrastive learning

In this work, the application of self-supervised contrastive learning (SSCL) [18] was explored in the context of histopathology image classification. SSCL is a promising approach that enables learning salient features from a limited amount of labeled data, making it particularly relevant when faced with a scarcity of labeled medical-image datasets [19]. Although our dataset contains fully labeled samples, we acknowledged that this is not always the case in medical imaging, where datasets often lack complete annotations.

The objective of the work was to assess the performance of Self-Supervised Contrastive Learning (SSCL) on our dataset, considering that only 16% of total the data was labeled. The training data and the testing data were constituted with 80% and 20% of the total data for the learning processes. The methodology proposed in the work considered only 20% random samples of the training data as labeled training data to train our model while considering the remaining 80% training data as unlabeled. Gradually, the labeled training data was augmented by incorporating the unlabeled data with pseudo labels generated by the recently trained model, with a high confidence threshold.

Initially, a pre-trained ResNet50 model was trained with the labeled training data. Following the initial training, pseudo labels were gradually generated for the unlabeled training data by the recently trained model. The pseudo-labels with confidence beyond 0.90 were assigned to the corresponding unlabeled samples and added the assigned pseudo-labeled samples to the labeled training data. The confidence threshold was kept as high as above 0.90 so that the labeled training data – when extended – does not get polluted with false data. The combination of the assigned pseudo labeled samples with the labeled training data was considered as the newly labeled training data to train the model in the following training epoch. Finally, the model's performance was evaluated on the testing data.

Algorithm 1 describes the whole process of Self-Supervised Contrastive Learning. The *mixUp* and *standard* functions mentioned in the algorithm are used to augment the processed data. The augmented data is split into training and testing data using the function *split*. The *randomSample* function is used to generate the initial labeled training data sampled randomly from the training data. The function *Train* handles the training process of the model with the labeled training data. The labels for the unlabeled are generated by the function *PredictLabels*. Depending on the confidence associated with the label generation, the extension of the labeled training data is filtered by the function *filterData*. The function *getAccuracy* determines the accuracy of the trained model on the testing data.

**Algorithm 1:**
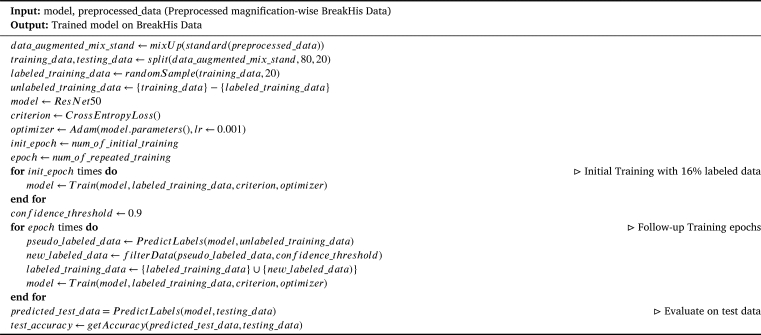
Algorithm for self-supervised contrastive learning.

#### 2.2.2 Transfer learning

Transfer learning is a machine learning approach that involves utilizing the knowledge and insights gained from training a model on one task to improve the performance of the model on a different but related task, thus saving time and resources in training from scratch. In this study, ResNet50, as detailed in [17], was employed as the foundational model for transfer learning. ResNet50 is a widely recognized convolutional neural network (CNN) architecture celebrated for its effectiveness in image classification tasks. Notably, it effectively mitigates the vanishing gradient problem through the implementation of skip connections, facilitating the direct flow of gradients to earlier layers. By learning residual mappings, ResNet50 adeptly captures disparities between input data and the desired output, thereby enhancing the efficiency of training and mitigating performance degradation in deep networks. Leveraging pre-trained weights on extensive datasets, ResNet50 serves as a robust foundational model for specific image classification tasks.

#### 2.2.3 Proposed architecture

In this study, the objective was to harness the skip connections within the ResNet50 architecture to craft a more streamlined model. The underlying aim was to strike a balance between model simplicity and computational efficiency while leveraging the skip connections to retain essential input information throughout the training process.

**Figure 2:**
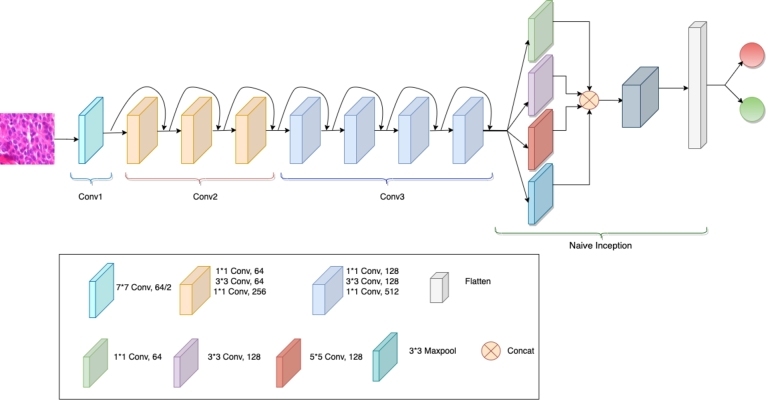
The proposed architecture, a fusion of Shallow ResNet50 and Naive Inception, follows a sequential arrangement of convolution layers labeled as conv1, conv2, and conv3. Additionally, the architecture incorporates a Naive Inception module, featuring parallel 1 × 1, 3 × 3, and 5 × 5 convolution layers, alongside a 3 × 3 maxpool layer. The resulting outputs from these layers are concatenated and directed to a dense layer for the final class prediction.

To formulate the lighter architecture, the initial blocks of ResNet were extended by the introduction of a novel block, while the final two sets of blocks (conv4 and conv5) were excluded, as visualized in Fig. 2. This newly introduced block, denoted as “Naive Inception,” concurrently processes the output of the Shallow ResNet50. Following this, the convolution outputs from each branch are concatenated, and a Dense layer is subsequently applied to generate the final output.

The Inception module, originally introduced in GoogLeNet [20], has demonstrated its proficiency in capturing diverse and multi-scale features through the utilization of parallel convolutional branches. A simplified version of the Inception module was incorporated into our architecture. It is important that our proposed model exhibits a substantially reduced parameter count compared to the ResNet50 architecture, as detailed in Table 2, and its architectural representation is depicted in Fig. 2.

**Table 2:** Architectures and number of parameters.

Architecture	#parameters
ResNet50	23,788,418
**Table:** Shallow ResNet50 + Naive Inception	3,649,506

Various combinations of freezing and fine-tuning techniques were employed in this study, tailored to different layers. Initially, the decision was made to freeze the initial blocks of ResNet to retain the pretrained weights. Following this, the newly introduced Naive Inception block and Dense layer underwent fine-tuning. Additionally, alternative configurations were explored, which encompassed freezing the entire ResNet and progressively unfreezing specific layers during the training process. These diverse strategies provided a means to capitalize on transfer learning while tailoring the model to suit the requirements of our image classification task. The following combinations were implemented, with block names corresponding to the labeled blocks in Fig. 2.

• Config. 1: Preserve all ResNet blocks. Train only Naive Inception module and Dense Layer
• Config. 2: Preserve Conv1, Conv2. Train Conv3, Naive Inception, Dense
• Config. 3: Preserve Conv1. Train Conv2, Conv3, Naive Inception, Dense
• Config. 4: Train the whole network

## 3 Results

In this work, a thorough evaluation of the proposed approaches for breast cancer detection and classification using histopathological images was performed. Self-supervised contrastive learning and Transfer learning techniques were employed, along with data augmentation and fine-tuning at various magnification levels. The results demonstrated improved accuracy in the identification and classification of malignant and benign breast cancer, underscoring the effectiveness of the methods employed.

### 3.1 Contrastive learning

A pretrained ResNet50 model was employed along with a cross-entropy loss function and the Adam optimizer. The model underwent 11 epochs of training, utilizing a batch size of 16 and a learning rate set at 0.001. These hyperparameters were selected with care to facilitate effective learning. A unique aspect of this approach was the incremental increase in the training data after each epoch. Samples that received high-confidence predictions in the previous epoch were systematically incorporated into the training data for subsequent epochs. This strategy enabled a gradual inclusion of additional labeled data, ultimately leading to enhanced model performance. Table 3 shows the training and test performance after each epoch. Test accuracies of 93.48% for 40X, 94.96% for 100X, 91.56% for 200X, and 93.68% for 400X magnified images were achieved.

**Table 3:**
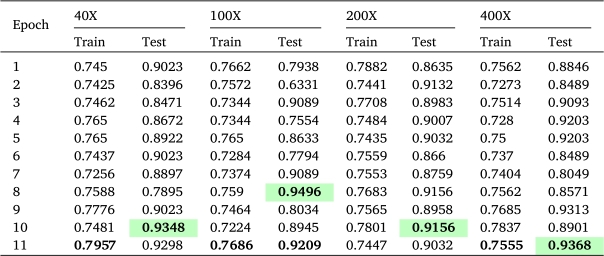
Contrastive learning performance (training and test accuracy).

### 3.2 Transfer learning

**Figure 3:**
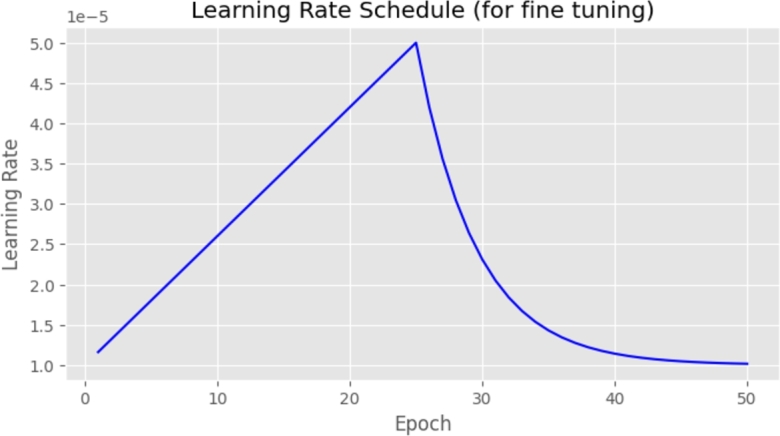
Learning rate scheduler.

A batch size of 128 was employed, along with the Adam optimizer set at a learning rate of 0.001, and categorical cross-entropy served as the chosen loss function. Each configuration underwent 50 epochs of training. For the fine-tuning phase, a learning rate scheduler was utilized, covering values within the range of $1\times 10^{-5}$ to $5\times 10^{-5}$ with a decay rate of 0.8. The scheduler is shown in Fig. 3.

**Figure 4:**
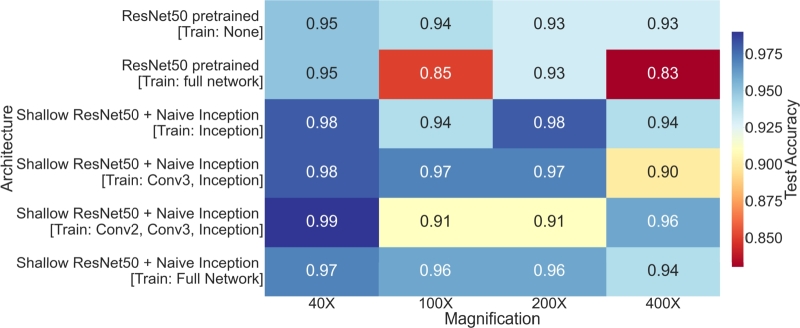
Performance of models with different configurations.

Fig. 4 illustrates the performance comparison between the ResNet and the proposed architecture across different configurations. The accuracy and F1-score on an undisclosed test dataset are displayed. This clearly demonstrates that the lightweight architecture consistently outperforms the ResNet50 architecture in all scenarios. Different trained models emerged as the top performers on our test dataset across various magnification levels of images. Specifically, for 40X images, configuration-3 of the training achieved the highest accuracy and F1-score, both at 0.99. Configuration-4 yielded a 0.96 accuracy for 100X images, while configuration-3 resulted in the same accuracy for 400X images. For 200X images, configuration-1 demonstrated an accuracy and F1-score of 0.98. In summary, configuration-1 exhibited comparatively superior performance across all magnification levels of images.

**Figure 5:**
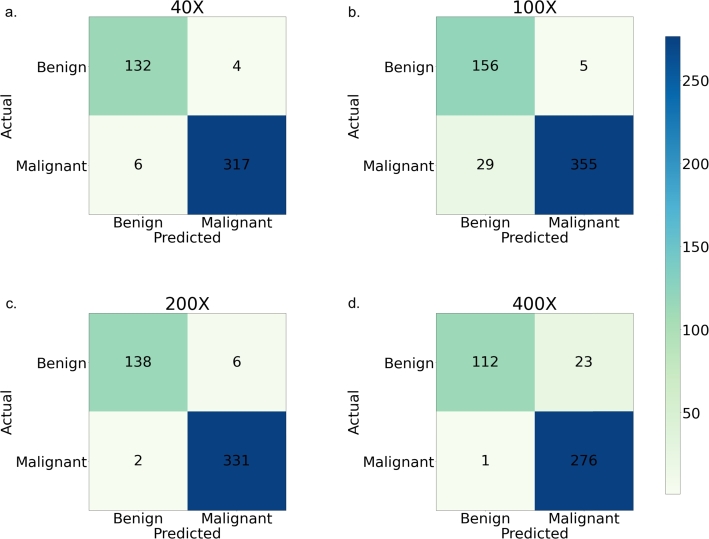
Confusion matrices for the architecture - Config 1. a. At 40X magnification, only 10 images are misclassified out of 459, showcasing high accuracy. b. For 100X magnified images, 34 instances are misclassified out of 545. c. represents the confusion matrix for 200X magnified images, revealing a remarkable accuracy with only 8 misclassifications out of 477. d. Lastly, at 400X magnification, 24 images are misclassified out of 412, offering a comprehensive overview of the model's classification efficacy across varying magnification levels.

**Figure 6:**
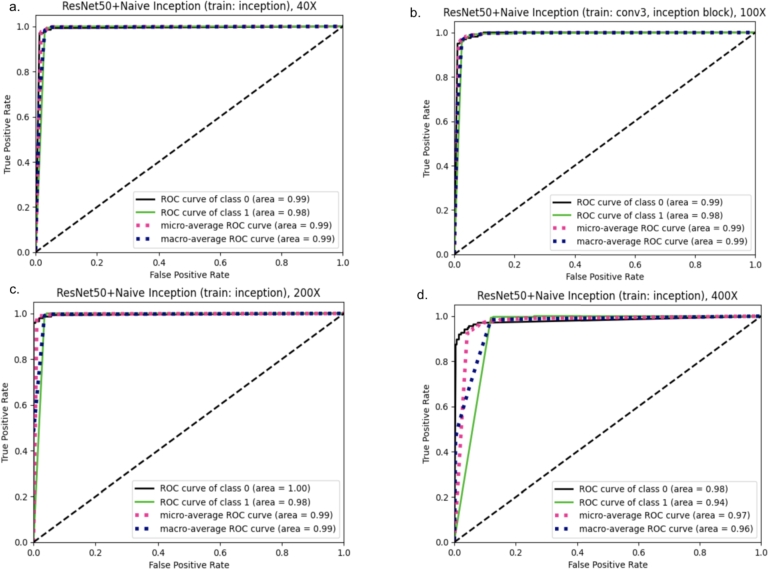
ROC-AUC curve for the fine-tuned architectures (Config 1) on a. 40X magnified images, b. 100X magnified images, c. 200X magnified images, d. 400X magnified images.

Fig. 5(a-d) displays the confusion matrix of the proposed architecture, which retains the ResNet part while training the Inception module, across various magnification levels of images. It is evident that the model exhibits outstanding performance in the classification of cancer images. Nevertheless, for 100X images, this model occasionally produces false negatives. In Fig. 6(a-d), the ROC-AUC plot for the model is presented. The AUC values are notably high, exceeding 0.98, for all classes in 40X, 100X, and 200X magnification levels. However, the AUC value is comparatively lower, at 0.94, for 400X images in the case of cancer images.

**Figure 7:**
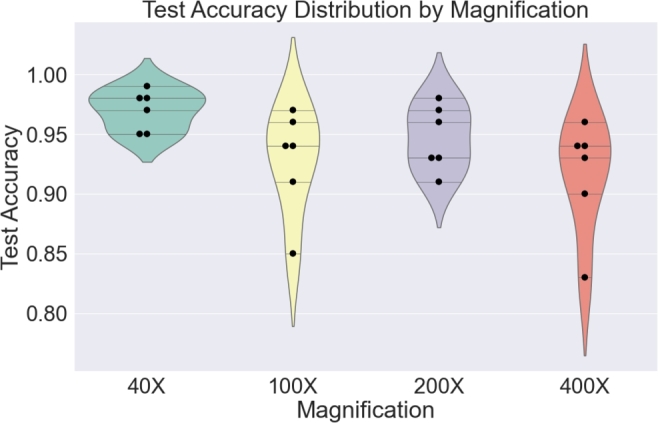
Performance vs. magnification.

Furthermore, an analysis of the impact of magnification levels on diagnosis accuracy was conducted. Fig. 7 illustrates the accuracies for each architecture at various magnification levels. It is evident that 40X and 200X magnified images are more conducive to accurate diagnosis, as the performance remains relatively consistent across different architectures. Conversely, 100X and 400X magnified images pose challenges for classification by all architectures, as indicated by the narrow and tall violin plots.

### 3.3 Comparison with existing works

A performance comparison has been undertaken with existing works, as outlined in Table 4. The table provides a summary of the accuracies achieved by various recent works, encompassing our proposed approaches. Observations indicate that the Custom CNN consistently maintained an accuracy range between 0.82 and 0.84 across various magnifications (40X, 100X, 200X, and 400X) [12], [13]. Many of the examined works achieved accuracies close to 0.90. Notably, the VGG16+Inception architecture attained approximately 0.97 accuracy for both 40X and 100X magnifications [16]. Additionally, the separately trained ResNeXt models demonstrated accuracies of 0.97 for 100X and 0.98 for 400X in the benign class [21]. In contrast, our proposed architecture [Shallow ResNet+Inception] achieved an accuracy of 0.98 for both 40X and 200X images, while obtaining 0.94 accuracy for 100X and 400X.

**Table 4:**
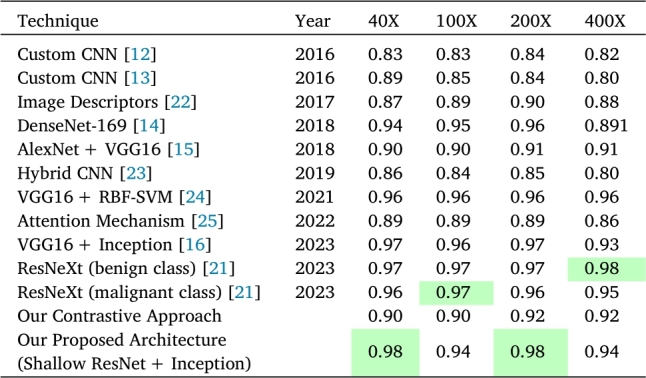
Comparison with existing works. See [12], [13], [22], [14], [15], [23], [24], [25], [16], [21].

## 4 Discussion

This work introduces a methodology involving the fine-tuning of the ResNet50 model, yielding promising outcomes. It is noteworthy that our approach demonstrates substantial enhancements in accuracy relative to prior research, attaining a remarkable accuracy rate of 0.98 at 40X and 200X magnifications. Previously, the highest recorded performance stood at 0.97 and 0.96 for distinct models using ResNeXt architecture, corresponding to the benign and malignant classes, respectively [21]. However, VGG16 architecture with Inception head also performed with similar accuracy for these magnification levels [16].

In the context of 100X magnification, it's worth noting that the highest reported accuracy, as per [21], stands at 0.97 for models trained separately on malignant and benign data. Our model, while achieving a slightly lower accuracy of 0.94, distinguishes itself by being a unified, single-trained model encompassing both classes. For 400X magnification, our model attains an accuracy of 0.94, whereas the class-specific models achieve 0.98 and 0.95 for the benign and malignant classes, respectively.

It is noteworthy that the models in question possess approximately 10.86 million parameters for the malignant class and approximately 13.46 million parameters for the benign class, as reported in [21], and roughly 8 million parameters in [16]. In contrast, our model is characterized by a significantly lower parameter count, standing at approximately 3.6 million parameters. This substantial reduction in parameter count in our model not only demonstrates its efficiency but also signifies its potential for streamlined implementation and reduced computational overhead, making it a practical and resource-efficient choice for breast cancer identification from histopathology image.

The trained model, characterized by a reduced parameter count, demonstrates exceptional performance when applied to histopathological images magnified at 40X and 200X. However, it falls short of achieving comparable results when dealing with images magnified at 100X and 400X. One plausible explanation for this discrepancy may lie in the limitations imposed by the model's reduced parameterization, hindering its ability to effectively capture features from images at these magnifications. This study underscores the potential of contrastive learning for feature extraction under data constraints, but it is essential to acknowledge that the effectiveness of proposed contrastive approach is contingent upon the selection of an appropriate model for small dataset learning.

## 5 Conclusions

Throughout this study, the primary objective involved the development of an efficient deep-learning-based classifier for Breast Cancer Histopathological Images. The study's focus remained centered on the optimization of the learning approach and refinement of the architecture to enhance overall performance. In this study, self-supervised contrastive learning was experimented as the designated learning approach, while a hybrid architecture consisting of Shallow ResNet50 and Inception module was utilized. The results demonstrate a state-of-the-art performance, achieving 98% accuracy for images magnified at 40X and 200X. Furthermore, the proposed architecture features a notably reduced parameter count, approximately 3.6M parameters, in contrast to the higher parameter counts observed in existing best architectures, approximately 8M [16] and approximately 10M + 13M [21].

In the context of histopathology image classification, researchers can draw valuable insights from our study. Firstly, they can adapt pre-trained models like ResNet50 through fine-tuning to address specific tasks, particularly in cases with limited or domain-specific labeled data. Additionally, customizing the model architecture by incorporating simplified modules, such as Inception, enables the efficient capture of diverse features while maintaining computational efficiency. It is crucial to consider the influence of image magnification levels on classification accuracy, allowing for the optimization of strategies tailored to different imaging scales and resolutions. Lastly, researchers can strike a balance between leveraging pre-trained knowledge and adapting the model to the dataset by employing selective customization, achieved through the fine-tuning of specific layers.

The achieved results in this study make significant contributions to the field of histopathology image classification. Firstly, the achievement of a state-of-the-art performance for images magnified at 40X and 200X is particularly promising, particularly in its potential applications for early diagnosis and treatment planning. Secondly, the hybrid architecture with a substantially lower number of parameters signifies computational efficiency and addresses resource constraints, making the model more accessible and feasible for deployment. In summary, the outcomes of this study not only advance the current state of histopathology image classification but also hold practical implications for enhancing the accuracy and efficiency of breast cancer diagnosis. This contribution contributes to the overall progression of medical image analysis within the healthcare domain.

## Ethics statement

Review and/or approval by an ethics committee was not needed for this study because the study is based entirely on computational analysis and does not involve human subjects or animal experimentation.

## CRediT authorship contribution statement

**Faisal Bin Ashraf:** Conceptualization, Formal analysis, Methodology, Writing – review & editing, Investigation, Visualization, Writing – original draft. **S.M. Maksudul Alam:** Conceptualization, Data curation, Investigation, Methodology, Writing – original draft. **Shahriar M. Sakib:** Data curation, Investigation, Validation, Visualization, Writing – original draft.

## Declaration of Competing Interest

The authors declare that they have no known competing financial interests or personal relationships that could have appeared to influence the work reported in this paper.

## Data Availability

Data associated with your study has not been deposited into a publicly available repository. The data associated with our study has been obtained from a publicly available dataset, the Breast Cancer Histopathological Database [BreakHis], which can be accessed at the following link: https://web.inf.ufpr.br/vri/databases/breast-cancer-histopathological-database-breakhis/. Our implementation and results are available in the GitHub repository at the following link: https://github.com/fbabd/Histopathological-image-breast-cancer-classification.

## References

[1] Azamjah N., Soltan-Zadeh Y., Zayeri F. (2019). Global trend of breast cancer mortality rate: a 25-year study. Asian Pac. J. Cancer Prev..

[2] Spanhol F.A., Oliveira L.S., Petitjean C., Heutte L. (2015). A dataset for breast cancer histopathological image classification. IEEE Trans. Biomed. Eng..

[3] Loukas C., Kostopoulos S., Tanoglidi A., Glotsos D.T., Sfikas K., Cavouras D.A. (2013). Breast cancer characterization based on image classification of tissue sections visualized under low magnification. Comput. Math. Methods Med..

[4] Peikari M., Gangeh M.J., Zubovits J., Clarke G., Martel A.L. (2015). Triaging diagnostically relevant regions from pathology whole slides of breast cancer: a texture based approach. IEEE Trans. Med. Imaging.

[5] Sahasrabudhe M., Christodoulidis S., Salgado R., Michiels S., Loi S., André F., Paragios N., Vakalopoulou M. (2020). Medical Image Computing and Computer Assisted Intervention–MICCAI 2020: 23rd International Conference, Lima, Peru, October 4–8, 2020, Proceedings, Part V 23.

[6] He L., Long L.R., Antani S., Thoma G. (2010). Computer assisted diagnosis in histopathology. Seq. Genome Analysis Methods Appl..

[7] He L., Long L.R., Antani S., Thoma G.R. (2012). Histology image analysis for carcinoma detection and grading. Comput. Methods Programs Biomed..

[8] Cheng H.-D., Shan J., Ju W., Guo Y., Zhang L. (2010). Automated breast cancer detection and classification using ultrasound images: a survey. Pattern Recognit..

[9] Abdel-Hamid O., Mohamed A.-r., Jiang H., Deng L., Penn G., Yu D. (2014). Convolutional neural networks for speech recognition. IEEE/ACM Trans. Audio Speech Lang. Process..

[10] Szegedy C., Liu W., Jia Y., Sermanet P., Reed S., Anguelov D., Erhan D., Vanhoucke V., Rabinovich A. (2015). Proceedings of the IEEE Conference on Computer Vision and Pattern Recognition.

[11] Ferrari A., Lombardi S., Signoroni A. (2017). Bacterial colony counting with convolutional neural networks in digital microbiology imaging. Pattern Recognit..

[12] Bayramoglu N., Kannala J., Heikkilä J. (2016). 2016 23rd International Conference on Pattern Recognition (ICPR).

[13] Spanhol F.A., Oliveira L.S., Petitjean C., Heutte L. (2016). 2016 International Joint Conference on Neural Networks (IJCNN).

[14] Gupta V., Bhavsar A. (2018). Proceedings of the IEEE Conference on Computer Vision and Pattern Recognition Workshops.

[15] Deniz E., Şengür A., Kadiroğlu Z., Guo Y., Bajaj V., Budak Ü. (2018). Transfer learning based histopathologic image classification for breast cancer detection. Health Inf. Sci. Syst..

[16] Saini M., Susan S. (2023). Vggin-net: deep transfer network for imbalanced breast cancer dataset. IEEE/ACM Trans. Comput. Biol. Bioinform..

[17] He K., Zhang X., Ren S., Sun J. (2016). Proceedings of the IEEE Conference on Computer Vision and Pattern Recognition.

[18] Bastanlar Y., Orhan S. (2022). Artificial Intelligence Annual.

[19] Ciga O., Xu T., Martel A.L. (2022). Machine Learning with Applications.

[20] Goodfellow I.J., Shlens J., Szegedy C. (2014). Explaining and harnessing adversarial examples. https://arxiv.org/abs/1412.6572.

[21] Sepahvand M., Abdali-Mohammadi F. (2023). Joint learning method with teacher–student knowledge distillation for on-device breast cancer image classification. Comput. Biol. Med..

[22] Gupta V., Bhavsar A. (2017). Proceedings of the IEEE Conference on Computer Vision and Pattern Recognition Workshops.

[23] Zhu C., Song F., Wang Y., Dong H., Guo Y., Liu J. (2019). Breast cancer histopathology image classification through assembling multiple compact CNNs. BMC Med. Inform. Decis. Mak..

[24] Albashish D., Al-Sayyed R., Abdullah A., Ryalat M.H., Almansour N. Ahmad (2021). 2021 International Conference on Information Technology (ICIT).

[25] Seo H., Brand L., Barco L.S., Wang H. (2022). Scaling multi-instance support vector machine to breast cancer detection on the breakhis dataset. Bioinformatics.

